# Exploring Early Childhood Autism Spectrum Disorders: A Comprehensive Review of Diagnostic Approaches in Young Children

**DOI:** 10.7759/cureus.50111

**Published:** 2023-12-07

**Authors:** Ruba A Alrehaili, Reem M ElKady, Jumana A Alrehaili, Reem M Alreefi

**Affiliations:** 1 Family Medicine, Academy of Family Medicine, Ministry of Health, Al-Madinah, SAU; 2 Radiology and Medical Imaging, Faculty of Medicine, Taibah University, Al-Madinah, SAU; 3 College of Medicine, Taibah University, Al-Madinah, SAU

**Keywords:** literature review, screening assessment, biomedical investigations, diagnostic instruments, diagnostic criteria, behavioral problems, communication, socialization, developmental disabilities, autism spectrum disorders

## Abstract

Autism spectrum disorders (ASD) encompass a diverse range of developmental disabilities characterized by pervasive deficiencies in socialization, communication, and the manifestation of behavioral issues. This review aims to clarify the diagnostic criteria for ASD, explore available diagnostic tools, evaluate biomedical examinations facilitating ASD diagnosis, and discuss the approach to screening assessments for ASD. ASDs are prevalent conditions, with a globally estimated prevalence of 7.6 cases per 100 (one in 132), based on a comprehensive review of epidemiological studies. The precise cause of autism and other ASDs remains unknown; however, genetic and environmental factors likely contribute. Common signs and symptoms shared among individuals with autism include restricted repetitive behaviors and learning disabilities. Standardized methods, such as the Diagnostic and Statistical Manual, Fifth Edition (DSM-5), diagnostic instruments, a thorough medical history, multiple physical examinations, laboratory investigations, neuroimaging, and screening tests, play a pivotal role in early autism diagnosis and intervention. ASD is a lifelong neurodevelopmental condition characterized by multiple deficits. Early detection is anticipated to have a positive impact on outcomes.

## Introduction and background

Autism spectrum disorders (ASD) constitute a complex neurodevelopmental disorder influenced by a spectrum of genetic, pathophysiological, and environmental factors, typically manifesting early in childhood and persisting throughout life [1]. The prevalence of ASD among school-aged children has surged from one in 150 in 2000 to one in 54 in 2016, highlighting its relatively common occurrence [2].

ASD represents a cluster of developmental disabilities characterized by deficits in social communication and the presence of restricted or repetitive interests and behaviors, aligning with the diagnostic criteria outlined in the Diagnostic and Statistical Manual, Fifth Edition (DSM-5) [3]. Research has consistently shown that children diagnosed with ASD often exhibit behavioral problems, including hyperactivity, poor attention, impulsivity, aggression, self-injury, and challenges in understanding social cues such as body language, gestures, and facial expressions. Moreover, these children frequently display unusual responses to sensory stimuli, such as hypersensitivities to light, sound, color, smell, or touch, and may demonstrate a heightened pain threshold [4].

Additionally, children with ASD manifest qualitative abnormalities in motor performance, which, although not part of the primary diagnostic criteria are believed to contribute to their clinical presentation and overall challenges [5,6]. Typically, children with ASD require substantial levels of supervised care, specialized educational resources, and healthcare services [7].

The diagnosis of ASD usually takes place in early childhood through behavioral observations, clinical presentations, or various imaging techniques such as functional MRI and diffusion tensor imaging. Imaging techniques often reveal abnormalities in volume associated with structural connectivity and atypical functionality in the brain. Magnetic resonance spectroscopy (MRS), a method allowing the quantification of various brain metabolites, including N-acetyl-aspartate (NAA), has reported a significant decrease in NAA concentrations in patients with ASD [8].

ASD necessitates a comprehensive, systematic, and structured approach that extends beyond the mere identification of the disorder. The diagnostic assessment provides invaluable insights into the child's strengths and weaknesses, laying the foundation for a well-informed roadmap for treatment [9]. The significance of an early and reliable ASD diagnosis cannot be overstated, as prompt interventions are essential once signs manifest.

## Review

Definition

ASD is a complex neurodevelopmental disorder characterized by three primary features: speech delay, challenges in social interaction and communication, and the presence of repetitive behaviors or desires [10]. Beyond these primary symptoms, autistic children may exhibit comorbidities such as learning disabilities, neurological conditions, depression, digestive deficiencies, and insomnia. In 1943, Kanner introduced the term "autism" for the first time [11]. Over the years, the diagnostic framework has undergone expansion to encompass various manifestations of autism. ASD includes autism, Asperger's disorder, autistic disorder, childhood disintegrative disorder, adolescent autism, and general developmental disorder not otherwise specified.

Epidemiology

The prevalence of ASD varies globally, ranging from 0.02% to 3.66% [12]. There is a higher frequency observed in males than females [13].

In different continents, the prevalence of ASD differs, with rates of 0.4% in Asia, 1% in America, 0.5% in Europe, 1% in Africa, and 1.7% in Australia [12]. The Gulf Cooperation Council (GCC) countries show an increasing prevalence of ASD, considering it one of the most common impairments [14]. Specific rates include 29/10,000 in the UAE, 1.4/10,000 in Oman, and 4.3/10,000 in Bahrain [14]. In 2002, Saudi Arabia identified 42,500 cases of autism, along with additional cases that went undiagnosed [15].

A recent systematic review disclosed that the prevalence of ASD in Arabian Gulf countries, including Saudi Arabia, ranged from 1.4 to 29 cases per 10,000 population, notably lower than the rates reported in developed countries (39 to 77 cases per 10,000) due to underdiagnosis [16]. A cross-sectional study involving 205 individuals diagnosed with ASD revealed a male-to-female ratio of 4.9:1. Comorbidities were present in 65% of patients, with attention-deficit hyperactivity disorder (ADHD) being the most prevalent (53%), followed by intellectual disability (8%), epilepsy (2%), and cerebral palsy (2%) [17].

Etiology

The specific cause of ASD remains uncertain, and hypotheses about its origins have evolved over time. While a psychosocial hypothesis linking autism to defective childbearing was once considered, it has been firmly rejected. Current evidence strongly supports the idea that the etiology of ASD is multifactorial, primarily rooted in genetics [18,19].

Family studies emphasize the familial and heritable nature of autism, with siblings of autistic children having a recurrence ratio ranging from 2% to 8%, surpassing that of the general population [20,21]. Twin studies reveal a higher concordance rate in monozygotic twins (90%) compared to dizygotic twins (10%) [21,22]. Previous research has explored the genetic underpinnings of autism through the identification of gene mutations or copy number variations at specific chromosomal locations associated with neurodevelopmental processes in individuals and families [23,24]. Noteworthy genes linked to the causation of autism, such as *FOXP2*, *RAY1/ST7*, *IMMP2L*, and *RELN* at 7q22-q33, have been recognized [25]. A study conducted by the International Molecular Genetic Study of Autism Consortium, encompassing 99 multiplex families, identified regions related to ASD on six different chromosomes, with chromosome 7 holding particular significance [26].

Various environmental factors are recognized as potential contributors to autism. Epidemiological studies point to prenatal viral infections, including measles, mumps, rubella, chicken pox, varicella-zoster, and cytomegalovirus infections, as contributing factors [27]. Cytomegalovirus, particularly, has been identified as a causative agent leading to enduring neurological impairment, affecting around 10%-20% of newborns when the mother experiences infection during pregnancy [27].

The correlation between maternal age, paternal age, and autism risk has been identified as a significant factor in several studies [28,29]. Sandin et al., in a meta-analysis, found a lower risk for autism (relative risk of 0.76) for mothers under 20 compared to those aged 25-29. Conversely, mothers aged 35 or older had a higher relative risk (1.52) compared to mothers aged 25-29 [30]. Reichenberg et al., in a study based on the population, indicated that the likelihood of autism starts to increase at the age of 30 and continues to rise after reaching the age of 50 [29]. Furthermore, zinc deficiency has been implicated in certain cases, where prolonged deficiency during pregnancy may result in various dysfunctions in embryonic growth, particularly in neurodevelopment [31]. Initial exploration into the relationship between zinc and autism originated from its association with neurodegeneration and dysfunction [32]. Recent studies also propose that the increased risk of ASD is associated with both the uptake of toxic metals and deficiencies in essential elements [33]. Notably, zinc has been observed to interact with β-amyloid and its precursors, which are crucial factors in the brain's degenerative processes [32].

Symptoms and signs of ASD

Social Communication

The early symptoms of ASD often manifest as delays and difficulties in social interaction, yet these signs may be subtle and easily overlooked. The absence of mutual attention, characterized by a failure to demonstrate interest and share a focus of attention, is particularly indicative of ASD [34]. Caregivers should be attentive to the child's consistent lack of response to their name, as this may suggest potential hearing impairment or ASD.

Additionally, stereotypical behaviors, such as insufficient facial expressions, the lack of a social smile, and limited use of gestures (e.g., clapping, smiling, shaking the head, nodding), are frequently observed. Children with ASD may exhibit a diminished understanding of the emotions of others and may be less attuned to the impact of their own attitudes on those around them [35].

Restricted, Repetitive Behaviors, Interests, and Activities

Restricted, repetitive behaviors, interests, and activities are commonly observed in children with ASD. These behaviors may include motor attitudes such as twirling, finger flicking, hand flapping, and headbanging [34]. It is widely acknowledged that children with ASD often engage in the repetitive use of items, such as lining up toys, and the repetitive use of words, specifically stereometric words with constant shape or pattern, as well as restricted echolalia. The term "restricted echolalia" refers to the specific imitation or reproduction of speech, that is repeated shortly after it has been heard [35].

Learning Disabilities

Historically, individuals with severe learning disabilities (defined as an IQ less than 70) were predominantly identified as having autism. Within the spectrum continuum, comorbid learning disorders are estimated to affect nearly 50% of individuals with ASD. These individuals may exhibit an irregular cognitive profile, characterized by significant variations between verbal and non-verbal scores in either direction. It is crucial to note, however, that individuals with "higher" scores in either verbal or non-verbal abilities may not necessarily reflect enhanced social skills or proficiency in daily coping life skills, which are likely to be severely affected [35].

Diagnostic criteria for ASD

Diagnostic criteria and severity level are shown in Table 1 [36].

**Table 1:** Diagnostic Criteria and Severity Levels for Autism Spectrum Disorder ASD, autism spectrum disorder.

Criteria	Symptoms	Severity Level for ASD
A. Persistent Deficits in Social Communication and Social Interaction		
1. Deficits in relationships	Challenges in adapting behavior to different social contexts, difficulties in engaging in imaginative play or forming friendships, lack of interest in interpersonal connections	Level 3: Requiring very substantial support
2. Deficits in social-emotional reciprocity	Abnormal social approaches, failure of normal back-and-forth conversation, reduced sharing of interests, emotions, or affect, inability to initiate or respond to social interactions	Level 2: Requiring substantial support
3. Deficits in nonverbal communicative behaviors	Poorly integrated verbal and nonverbal communication, abnormalities in eye contact and body language, deficits in understanding and using gestures, loss of facial expressions and nonverbal communication	Level 1: Requiring support
B. Restricted, Repetitive Patterns of Behavior, Interests, or Activities		
1. Insistence on sameness	Inflexible adherence to routines or ritualized patterns of behavior, extreme distress at minor changes, difficulties with transitions, rigid thinking patterns, greeting rituals, or a need for routine	Level 3: Requiring very substantial support
2. Stereotyped or repetitive behaviors	Simple motor stereotypes like lining up toys or flipping objects, engaging in echolalia and using idiosyncratic phrases, repetitive motor movements, use of objects, or speech	Level 2: Requiring substantial support
3. Hyper- or hyporeactivity to sensory input	Apparent indifference to pain or temperature, adverse responses to specific sounds or textures, excessive smelling or touching of objects, visual fascination with lights or movement	Level 1: Requiring support
4. Highly restricted and fixated interests	Abnormal intensity or focus, strong attachment to or preoccupation with unusual objects, excessively circumscribed or perseverative interests	Level 1: Requiring support
C. Symptoms in Early Childhood		
1. Early caregiver reports	No longer deemed essential	
2. Early childhood	Approximately age 8 and younger	
D. Symptoms Limit and Impair Everyday Functioning		
1. Minimal rituals and repetitive behaviors (RRBs) impairments	Significant difficulties with functioning due to RRBs, resistance to efforts to disrupt or divert RRBs from fixed interests	Social communication severity: Level 3, restricted interests severity: level 3
2. Minimal social impairment	Social communication deficiencies result in noticeable impairments, challenges in facilitating social encounters, and atypical or ineffective reactions to other people's social overtures	Social communication severity: level 1, restricted interests severity: level 1
E. Disturbances Not Better Explained		
Not better explained by intellectual disability or global developmental delay	Intellectual impairment and ASD may co-occur, and social communication may persist below the predicted average developmental level in comorbid cases	

Diagnostic instruments for ASD

Evaluation of a Child With ASD

The primary objective of the ASD evaluation is to identify effective strategies and care tailored to the child and family. This involves recognizing coexisting conditions and associated developmental issues that could negatively impact the patients and their families [37].

Diagnosing ASD can be challenging; the National Institute for Health and Care Excellence (NICE) offers recommendations on the assessment and treatment of ASD, urging clinicians in the United Kingdom to guarantee that children and adolescents under suspicion of ASD can avail the services of a regional multidisciplinary autism team. This team assumes a pivotal role in offering support and specialized knowledge in the evaluation and creation of profiles for individuals and their families [34].

History

ASD is identified through a thorough examination of developmental history and behavioral observation across settings like school, home, and social care. Documentation should encompass behavior duration, severity, and consequences. Despite consistent characteristics, their expression may vary in different environments. The analysis should consider the child's overall development, medical history, and potential risk factors [38].

Importantly, the absence of other symptoms and signs, such as poor eye contact or social smiling, does not rule out an ASD diagnosis. It is essential to acknowledge that the absence of parental apprehension regarding early development does not automatically imply a standard developmental trajectory [39].

Examination

As part of the evaluation, a comprehensive physical examination is necessary, incorporating a thorough neurological assessment, dysmorphism regulation, scrutiny for neurocutaneous stigma, and a Woods light (ultraviolet light) examination [40]. It is imperative to identify signs of illness, self-harm, and potential ill-treatment during this process. 

Laboratory Investigations

ASD is recognized as a developmental neurological disorder, prompting numerous studies to identify potential biomarkers associated with its development.

Elevated whole-blood serotonin, or hyperserotonemia, represents the first identified biomarker in ASD, present in over 25% of affected children [41]. The contribution of the serotonin system to ASD pathophysiology remains incompletely understood, with emerging data from neuroimaging and postmortem samples indicating changes in the brain serotonin system in ASD [41].

Unusual patterns of visual preference, as gauged by eye-tracking, are considered hallmarks; however, their utility as an early biomarker for ASD remains uncertain [42].

Melatonin deficit has been reported in several studies based on plasma or urine samples from individuals with ASD [43].

Identification of small-molecule peptides using blood plasma proteomic profiling and examination of cerebrospinal fluid can reveal modified patterns of proteins/peptides [33].

Proteomic analysis of serum from children with autism has revealed differential expression of apolipoproteins and other components of protein complements [43].

Magnetic Resonance Imaging (MRI)

Structural MRI:* *The literature on structural MRI in ASD provides significant evidence of volume differences in both gray and white matter, revealing distinct region-specific variations [44]. Examinations of the basal ganglia have revealed an increased size of the caudate in individuals with autism, and this enlargement corresponds with the intensity of repetitive behaviors [44]. Establishing a connection between neuroanatomical observations and behavioral manifestations is essential for understanding the impact of structural alterations on the origins of ASD.

A noteworthy discovery in the literature is the swift expansion of brain volume in early childhood, characterized by an approximately 10% rise, reaching its peak around 2-4 years [44]. This initial phase of accelerated growth is usually followed by a stabilization, evident in both gray and white matter. Research suggests a more pronounced association with the growth of white matter in early childhood, as indicated by meta-analyses conducted by Amaral et al. and Stanfield et al., proposing that age plays a crucial role and this type of enlargement is predominantly observed in young children with ASD [45,46].

The diversity of these observations emphasizes that autism affects both gray and white matter. Recent advancements in technology now allow for a more nuanced assessment of the composition of gray and white matter structures. Specifically, cortical gray matter can be precisely characterized in terms of cortical thickness and surface area, providing a collective estimate of cortical gray matter volume. Cortical thickness serves as an indicator of dendritic arborization and pruning within gray matter or alterations in myelination at the white-gray matter interface. Meanwhile, variations in surface area correspond with the extent of cortical folding or gyrification, believed to be influenced by the division of progenitor cells during embryogenesis in the periventricular region [44]. The analysis of variations in these measurements of cortical gray matter yields crucial insights into early neuroanatomic developmental events in the ASD population.

Functional magnetic resonance imaging (fMRI):* *fMRI studies uncover distinctive neuroanatomical patterns in ASD. Hyperactivation is observed in subcortical structures, encompassing the bilateral thalamus, bilateral caudate, and the right precuneus, particularly evident during emotional-face processing. In contrast, there is hypoactivation in the hypothalamus during the same emotional-face processing task. Sub-analyses with more homogeneous contrasts confirm and preserve the key findings of the main analysis, emphasizing the hyperactivation in subcortical structures [47].

Notably, abnormalities in subcortical structures, including the amygdala, hypothalamus, and basal ganglia, are intricately linked to atypical emotional-face processing observed in individuals with ASD [47]. These findings shed light on the intricate interplay between neuroanatomical structures and emotional processing in the context of ASD, advancing our understanding of the neural underpinnings of this complex developmental disorder.

Screening Recommendations

Studies have demonstrated that ASD can often be identified at or below 18 months of age, with a more accurate evaluation possible by age 2 [48]. However, conclusive diagnoses may not be reached until later stages, underscoring the potential delay in necessary care for children with ASD. Early diagnosis significantly influences the initiation of effective recovery programs. The American Academy of Pediatrics (AAP) recommends developmental screening at 9, 18, and 30 months during regular well-child visits. Additional screening may be necessary for children at elevated ASD risk or displaying signs, emphasizing the importance of screening all children, particularly those at higher risk due to factors such as preterm birth or having a family history of ASD [48].

Developmental Screening Tools

Various screening tools assist in identifying children with developmental delays, either disorder-specific or covering multiple areas. The CDC does not endorse specific tools, and the list is non-exhaustive, with options for pediatric practices, school systems, and community settings [48].

Types of screening tools: Several developmental screening tools exist, and selected examples include the following.

Communication and Symbolic Behavior Scales (CSBS): A standardized instrument assessing communication and symbolic skills up to the 24-month level. The Infant-Toddler Checklist is a screening tool completed by parents.

Ages and Stages Questionnaires (ASQ): A comprehensive developmental screening tool, consisting of a parent-completed questionnaire with sections tailored to specific age groups, screening across various domains.

Modified Checklist for Autism in Toddlers (M-CHAT): Parent-completed questionnaire identifying children at risk for autism in the general population.

Parents’ Evaluation of Developmental Status (PEDS): An overall developmental screening tool. It is a parent interview form designed to screen for developmental and behavioral issues, utilizing a single response form applicable to all age groups [49].

Prognosis

Behaviors and presentations exhibit temporal variations, showing a tendency for improvement across various domains, though substantial individual variability exists. A majority of individuals necessitate consistent and precise support throughout their lives. While many adults require ongoing full-time assistance, a small percentage of those with higher-functioning ASD (15%) may achieve independence and gain employment [43].

Critical factors influencing outcomes include the severity of behavioral manifestations, cognitive capacities, and verbal abilities. Researchers have reported promising outcomes for individuals undergoing early interventions that emphasize skill development. However, further research is imperative to ascertain whether these early interventions can exert a lasting impact on individuals with ASD [50].

Treatments and interventions

ASD therapies encompass a spectrum of medical, clinical, speech/language, behavioral, physical therapy, supportive, and alternative approaches to medication. In the treatment of children within the target age range for routine ASD screening, behavioral interventions, particularly early comprehensive behavioral and developmental treatments, play a predominant role. These interventions may employ strategies that integrate elements of cognitive-behavioral therapy, parent reinforcement components, and play or interaction-based approaches [50]. Among the various therapeutic approaches, those centered on integrated behavioral analysis present the highest-quality evidence showcasing their impact on cognitive and language outcomes. Implementation of such strategies may occur in a home or school environment, typically demanding a significant time commitment, with certain programs extending up to 40 hours a week [51].

Applied behavior analysis (ABA) is a treatment firmly rooted in learning theories and behavioral conditioning. It utilizes precise intervention objectives paired with positive reinforcement, including verbal praise, tokens, or rewards [52]. An essential element of ABA is the repetition of learning trials. A comprehensive meta-analysis assessing the efficacy of ABA interventions for young children diagnosed with autism demonstrated moderate to substantial positive impacts on intellectual functioning, language development, acquisition of daily living skills, and social functioning [53]. Significantly larger effect sizes were evident in outcomes related to language. Another noteworthy intervention is pivotal response treatment (PRT), which utilizes a more naturalistic behavioral approach focusing on particular skills and motivations, particularly pivotal areas [54]. In a randomized controlled trial, PRT exhibited advantages for functional and adaptive communication skills in 53 children (aged 2 to 6 years) diagnosed with autism and significant language delay [55]. In comparison to ABA, PRT was regarded as more effective in enhancing verbal expressive communication and associated with a diminished occurrence of disruptive behaviors.

Approximately 70% of individuals with autism also experience concurrent mental health conditions, including ADHD, irritability, aggression, as well as mood and anxiety issues [56]. Risperidone and aripiprazole have received FDA approval for addressing irritability associated with ASD, with risperidone approved for children aged at least five years and aripiprazole for those at least six years old [56]. Prolonged studies assessing the effectiveness of risperidone in ASD have demonstrated its efficacy in reducing behavioral symptoms and impulsivity compared to haloperidol. Additionally, a two-month randomized, double-blind, placebo-controlled trial found no significant differences between aripiprazole and risperidone in children and adolescents with ASD and associated behavioral symptoms. This evaluation considered primary outcome measures (ABC scores) and safety measures, including alterations in appetite and weight gain [57].

## Conclusions

ASD, characterized by behavioral challenges in social interactions and repetitive patterns of behavior, activities, and interests, is a heterogeneous and enduring neurodevelopmental disorder. The exact etiology of ASD remains elusive, yet evidence strongly points toward a significant hereditary influence. The diagnosis of suspected ASD in children involves a prompt and multidisciplinary procedure, with local pathways facilitating connections to specialized ASD teams. Various diagnostic modalities, including lab investigations and neuroimaging, contribute to the comprehensive assessment of ASD. Screening assumes a pivotal role in the early detection of ASD, with the anticipation that early diagnosis and early intervention can positively influence outcomes. Recognizing and effectively addressing associated morbid conditions is imperative for comprehensive care.
